# Improvement of hospital care for patients with non-Hodgkin’s lymphoma: protocol for a cluster randomized controlled trial (PEARL study)

**DOI:** 10.1186/1748-5908-8-77

**Published:** 2013-07-09

**Authors:** Jozette JC Stienen, Rosella PMG Hermens, Lianne Wennekes, Saskia AM van de Schans, Helena M Dekker, Nicole MA Blijlevens, Richard WM van der Maazen, Eddy MM Adang, Johan HJM van Krieken, Petronella B Ottevanger

**Affiliations:** 1 Scientific Institute for Quality of Healthcare (IQ healthcare), Radboud University Nijmegen Medical Centre, PO box 9101, 6500 HB Nijmegen the Netherlands; 2 Department of Radiology, Radboud University Nijmegen Medical Centre, PO box 9101, 6500 HB Nijmegen, the Netherlands; 3 Department of Hematology, Radboud University Nijmegen Medical Centre, PO box 9101, 6500 HB Nijmegen, the Netherlands; 4 Department of Radiotherapy, Radboud University Nijmegen Medical Centre, PO box 9101, 6500 HB Nijmegen, the Netherlands; 5 Department of Health Evidence, Radboud University Nijmegen Medical Centre, PO box 9101, 6500 HB Nijmegen, the Netherlands; 6 Department of Pathology, Radboud University Nijmegen Medical Centre, PO box 9101, 6500 HB Nijmegen, the Netherlands; 7 Department of Medical Oncology, Radboud University Nijmegen Medical Centre, PO box 9101, 6500 HB Nijmegen, the Netherlands; 8 Department of Registry and Research, Comprehensive Cancer Centre the Netherlands (CCC), PO Box 19079, 3501 DB Utrecht, the Netherlands

**Keywords:** non-Hodgkin’s lymphoma, Quality of healthcare, Guidelines, Oncology, Implementation, Interventions

## Abstract

**Background:**

Malignant lymphomas constitute a diverse group of cancers of lymphocytes. One well-known disease is Hodgkin’s lymphoma; the others are classified as non-Hodgkin’s lymphoma (NHL). NHLs are the most common hematologic neoplasms in adults worldwide, and in 2012 over 170,000 new cases were estimated in the United States and Europe.

In previous studies, several practice gaps in hospital care for patients with NHL have been identified. To decrease this variation in care, the present study aims to perform a problem analysis in which barriers to and facilitators for optimal NHL care will be identified and, based on these findings, to develop (tailored) improvement strategies. Subsequently, we will assess the effectiveness, feasibility and costs of the improvement strategies.

**Methods/design:**

Barriers and facilitators will be explored using the literature, using interviews and questionnaires among physicians involved in NHL care, and patients diagnosed with NHL. The results will be used to develop a tailored improvement strategy. A cluster randomized controlled trial involving 19 Dutch hospitals will be conducted. Hospitals will be randomized to receive either an improvement strategy tailored to the barriers and facilitators found or, a standard strategy of audit and feedback.

The effects of both strategies will be evaluated using previously developed quality indicators. Adherence to the indicators will be measured before and after the intervention period based on medical records from newly diagnosed NHL patients. To study the feasibility of both strategies, a process evaluation will be additionally performed. Data about exposure to the different elements of the strategies will be collected using questionnaires. Economic evaluation from a healthcare perspective will compare the two implementation strategies, where the costs of the implementation strategy and changes in healthcare consumption will be assessed.

**Discussion:**

The presence of variation in the use of diagnostic tests, treatment, and follow-up between different physicians in different hospitals in the Netherlands is important for patients. To reduce the existing variation in care, implementation of tailored interventions to improve NHL care is necessary.

**Trial registration:**

This trial is registered at ClinicalTrial.gov as the PEARL study, registration number NCT01562509.

## Background

Malignant lymphomas represent a heterogeneous group of malignant lymphocyte proliferations, which can be classified as either Hodgkin’s lymphoma (HL) or non-Hodgkin’s lymphoma (NHL). NHLs are the sixth most common malignant neoplasm in the United States [1] and the most common hematologic neoplasm in adults worldwide [2]. In 2012, the estimated number of new cases was 79,000 for the United States [1] and over 93,000 for Europe [3]. NHL constitute of more than 40 disease entities. The most prevalent are diffuse large B-cell lymphoma, follicular lymphoma, marginal zone cell lymphoma, small lymphocytic lymphoma and mantle cell lymphoma, together accounting for 80% of all NHL [4].

Evidence-based guidelines on diagnosis, treatment, and follow-up of patients with NHL have been developed during the past years and provide recommendations for high quality NHL care [5-11]. These national and international guidelines support physicians as well as patients in their decisions about diagnosis, therapy, and follow-up. In general, publication and distribution of these guidelines is not sufficient to maximize their effect on the quality of care [12], hence active implementation is needed. Several studies demonstrated suboptimal care for patients with NHL [13-15]. They described large gaps between daily practice and high-quality NHL care as recommended in the guideline. This lack of guideline adherence concerned, among others, diagnostics, therapy, and prognostic parameters. For example, in 22 Dutch hospitals, adherence was lowest for assessment of International Prognostic Index (IPI), documentation of indicator lesions found on CT-scans during diagnostics and after therapy, adequate pathology reporting, and discussing patients during multidisciplinary consultations [15]. The clinical relevance of guideline adherence is shown in several cancer studies [16-20]. These studies showed that better adherence to guidelines is associated with better overall survival or progression-free survival.

To improve quality of NHL care, it is important to know what the barriers to and facilitators for high-quality NHL care are. Based on this, tailored interventions can be developed to improve the quality of care. Most theories on implementation of evidence in healthcare emphasize assessment of influencing factors first, in order to acquire a tailor-made improvement strategy [21,22]. Several models have shown to be effective in structuring the experienced barriers and facilitators [23-25]. In the final steps, the strategies can be tested and evaluated.

### Aim and objectives

This study aims to perform a problem analysis in which barriers and facilitators for optimal NHL care will be identified. Based on these findings, we intend to develop (tailored) improvement strategies, and assess their effectiveness, feasibility and costs.

Main objectives that will be investigated are:

1. To explore barriers and facilitators according to patients and physicians, that influence optimal NHL care as described in evidence based NHL guidelines,

2. To gain insight into the current NHL care based on previously developed quality indicators.

3. To develop, test, and evaluate the improvement strategies, tailored to the barriers found and current practice.

## Methods

The objectives of this study will be examined in a cluster randomized controlled trial (cRCT), preceded by a problem analysis.

### Problem analysis

#### Design and methods

Barriers and facilitators for delivering optimal care will be investigated at different levels. For the classification of the influencing factors a framework developed by Cabana [23] and Grol [25] will be used. This framework includes features of the guidelines; features of the target group of physicians; features of patients; features of the social context (e.g., colleagues of the involved physicians and geographic distance to hospital); and features of the organizational context.

To detect possible barriers and facilitators regarding optimal NHL care, a literature study will be performed. Second, a qualitative study will be conducted on the basis of (small group) interviews among physicians involved in NHL care and on individual interviews with NHL patients. The interviewer will follow the framework as described above during the semi-structured interviews to explore influencing factors. This method will provide us with barriers and facilitators at different levels (e.g., patient, physician, as well as the organization). Finally, a questionnaire survey will be performed to quantify the features mentioned in the (small group) interviews. All participants will receive a web-based questionnaire by email and asked to fill in this questionnaire. The data will be gathered in an electronic database.

#### Study population and setting

Four small group interviews with seven to 10 physicians will be conducted. Physicians, including hematologists, pathologists, radiation oncologists, radiologists, and nuclear medicine physicians, from 22 hospitals involved in a previous NHL study [15] will be invited to participate in the interviews. These hospitals include university, teaching, and non-teaching hospitals. Patients will be invited to take part in the individual interviews by the Dutch Lymphoma Organization (LVN) and their attending physician. The interviews will be held with 15 to 20 patients until saturation has been reached. The questionnaire survey will be performed among 200 physicians and patients. To select the participants, we will contact the national professional associations of all professions involved and request e-mail addresses. Additionally, the LVN will be asked to invite patients via their website.

#### Outcome measures

Barriers and facilitators regarding optimal NHL care based on evidence-based multidisciplinary NHL guidelines. They will be classified within the framework developed by Cabana and Grol [23,25].

#### Data analysis

The barriers and facilitators mentioned in the interviews with physicians and patients will be qualitatively analyzed using the software Atlas.ti® (version 6.2.23, Atlas.ti Scientific Software Development GmbH; Berlin, Germany) and will be descriptive. Potential barriers and facilitators will be identified independently by two members of the project team, and any discrepancies will be discussed until consensus is reached.

The influencing factors from these interviews are quantified on the basis of questionnaire surveys conducted among physicians and patients to assess the most frequently mentioned barriers and facilitators. Analysis of the questionnaires will be descriptive (i.e., frequencies and means).

### Cluster randomized controlled trial

#### Design and methods

Based on the results of the problem analysis and the care measurements of Wennekes et al. [15], tailored interventions will be developed in order to increase indicator adherence. Current care will be assessed before start of the intervention period and the tailored interventions will be adjusted if necessary.

The interventions will be tested in a cRCT in 19 Dutch hospitals. The hospitals will be the level of randomization which includes two arms: centers receiving a standard strategy of audit and feedback, and centers receiving the standard strategy followed by an improvement strategy tailored to the barriers and facilitators found. Both strategies will be evaluated with an effect-, process-, and cost-evaluation. In the effect evaluation, the previously developed quality indicators will be used as effect measures: before and after implementing the strategies, quality indicator adherence will be assessed by retrospective searches in medical records by the Comprehensive Cancer Centre the Netherlands (CCC). After the intervention period, patients as well as physicians will be asked to fill out a questionnaire concerning the use and experiences with the interventions tested. Semi-structured interviews in each hospital will give insight into the processes concerning implementation of the interventions and local initiatives to improve NHL care during the intervention period. Costs of the interventions and the accompanied changes in consumption of care will be assessed as well.

#### Study population and setting

Nineteen hospitals, including university, teaching, and non-teaching hospitals, and their physicians involved in NHL care will participate in this study. Hospitals that participated in a previous NHL study [15] will be invited to take part in the current cRCT. An invitation letter will be send by email to the contact persons, accompanied by an informed consent.

Randomization will take place after formal agreement of all hospitals to participate in the trial and will be based on randomly generated numbers (computer based) stratified by hospital size (small, medium, and large). The contact persons of all participating hospitals will be informed of the allocation after collection of the baseline measurement. In all participating hospitals, the trial will be conducted between November 2012 and June 2014.

Per hospital, approximately 22 newly diagnosed NHL patients will be selected for data collection regarding adherence to the quality indicators. Selection of patients will take place using the cancer registry, with support of the CCC. The CCC will use the cancer registry to make a list of potentially eligible patients in the participating hospitals. From each list, the first 25 to 30 patients will be selected, and listed according to day of birth. This cancer registry is based on the pathology coding system of the World Health Organization (WHO) and patients with mature B-, T- and NK-cell neoplasms will be selected for inclusion. Patients diagnosed with cutaneous or leukemia-type neoplasms will be excluded, as well as patients younger than 18 years old.

During the intervention period, physicians of the intervention hospitals will hand out patient information and an informed consent to all newly diagnosed NHL patients. Physicians are asked to use the different developed elements to improve quality of care. Patients will return their informed consent with permission (or prohibition) for sending them a questionnaire at the end of the intervention period. Patients as well as physicians will be stimulated to actively apply the interventions of our improvement strategy (when applicable).

### Outcome measures

#### Effect measures

The effect evaluation aims to determine the effectiveness of the standard audit and feedback strategy versus the tailored improvement strategy using the previously developed quality indicators. Percentage of adherence to these indicators will be used as a primary measure for quality of NHL care. The effect measurement will be done by two assessments, one before and one after implementation of the improvement strategies. The data for these indicators will be collected on patient level from medical records. Additionally, morbidity in the patient groups, patient-related outcome measures, and potential confounders of the effects will be evaluated.

#### Process measures

To study the feasibility of both strategies, the process evaluation has to give insight into the mechanisms and processes responsible for the result (i.e., extent of adherence to the indicator set for optimal NHL care). The actual exposure of the patients and physicians to the interventions, together with their experience with these elements may have influenced the final result (success or failure of indicator adherence).

Data about exposure to the different interventions will be collected using a combination of data collection methods (e.g., user data of developed tools and questionnaires for physicians and patients). This information will be related to effectiveness of the strategy to assess which interventions were particularly associated with successful implementation. At the end of the intervention period, experiences of patients and physicians with the implementation elements will be measured with questionnaires and/or interviews. This information will be used to, if necessary, adapt the interventions to make them more acceptable and effective for implementation on a national level.

#### Cost measures

The economic evaluation will compare the two strategies in a healthcare perspective. Both the costs of the implementation strategy and changes in healthcare consumption will be assessed. Although the underlying technologies might be evaluated well on a national level, the aim of this analysis is to detect which strategy is the most cost-effective strategy on optimal care in common practice.

The input of resources in the improvement strategies will be assessed by collecting volumes of consumed resources and multiplying these by the price of each resource unit (market prices, guideline prices, or self-determined prices based on costing methods, i.e., full costing) [26,27]. The decision criterion on which the efficient implementation strategy will be selected is the incremental cost per gained percentage adherence.

#### Sample size considerations

We hope to detect a difference in adherence to the indicators between the two strategies of at least 20%. In a previous study, the mean adherence rate was 40%. To detect a difference of 20% (40% versus 60%), with alpha = 0.05, a two-sided testing and power = 0.80, at least 194 NHL patients are required.

However, considering an intra-cluster correlation coefficient of 0.09 (mean ICC in previous study [15]) and a hospital cluster correlation of 0.8, in 19 hospitals, 22 patients per hospital are needed (n = 418 patients).

### Data analysis

#### Effect evaluation

Multilevel regression analyses will be performed to evaluate the effectiveness of both improvement strategies. Patient characteristics as gender, age, tumor type, and co-morbidity will be collected and included in the analysis as possible confounders for differences in actual care between the different hospitals and for differences in effectiveness between the two strategies.

#### Process evaluation

Exposure to and experiences with the improvement strategy elements will be analyzed descriptively. Possible local initiatives to improve NHL care during the intervention period will also be considered descriptively.

#### Cost evaluation

The implementation process and consequently (full) costs of both strategies will be estimated by an Activity Based Costing (ABC) approach focusing on activities performed with costs accumulated at the activity level(s) of the healthcare implementation processes. The incremental costs will be determined by the difference in resource consumption between the two strategies. Next, the underlying activities (personnel, material, and overhead costs) associated with these sub-processes are identified. Use of ABC concepts facilitates the identification of non-value-added activities.

The output or consequences of the implementation strategies will be determined by the level of adherence to the quality indicators, measured before and after the intervention period. Adherence will be determined by using the repeated measures method over the two different time intervals. The decision criterion on which the efficient implementation strategy will be selected is the incremental cost per gained percentage adherence. The impact of variable(s) uncertainty on the decision criterion will be evaluated by sensitivity analyses.

#### Ethical considerations

In the Netherlands, studies involving human subjects need to undergo a medical ethics review if they are subject to the Medical Research Involving Human Subjects Act (WMO). The Medical Ethics Committee (CMO) of district Arnhem/Nijmegen assessed the study protocol and declared that no further ethical approval was required (registration number 2011/560). Therefore, using the described study protocol, the study will be carried out in accordance with the applicable rules concerning the review of research ethics committees and informed consent.

#### Trial status

No data cleaning or analysis of the cRCT has been executed prior to submission of this manuscript.

## Discussion

This paper describes the protocol of an improvement study, consisting of a problem analysis and a cRCT, to assess barriers and facilitators for optimal NHL care to develop, test, and evaluate (tailored) improvement strategies, based on these findings.

The strength of this study is the use of multifaceted tailored interventions, which may be more effective than single ones [22,28,29]. Therefore, a strategy with different interventions is supposed to be effective, directed at professional, patient, and organizational level. This strategy accompanies the disadvantage of measuring the effect of the entire package of interventions, which makes it unable to distinguish which intervention was most effective regarding improved NHL care (with indicator adherence as effect measure). During the process evaluation, we will try to get insight into the effectiveness of each separate intervention by acquiring information from participants concerning the exposure to, use of, and experiences with the different interventions.

Local initiatives to optimize NHL care during the intervention period might bias our study results. Therefore, information on local, non-study-related interventions will be additionally inquired during the process evaluation.

The results of this study will contribute to a better knowledge of barriers and facilitators of optimal NHL care and thus improvement opportunities. Next to this, insight will be obtained in the effects of different strategies on the quality of NHL care, which enables us to define a preferred improvement strategy. We believe that the interventions tailored to the barriers found will optimize NHL care and might be applicable on a national level in the future.

## Abbreviations

NHL: non-Hodgkin’s lymphoma; ZonMw: Netherlands organization for health research and development; LVN: Dutch lymphoma organization; cRCT: cluster randomized controlled trial; CCC: Comprehensive cancer centre the netherlands; WHO: World health organization; B- T- and NK-cell: B-lymphocyte, T-lymphocyte and natural killer cell; ICC: intra-cluster correlation coefficient; ABC: Activity based costing; WMO: Medical research involving human subjects act; CMO: Medical ethics committee.

## Competing interests

The authors declare that they have no competing interests.

## Authors’ contributions

RH and PO had the basic idea for this study. RH, PO, and JvK were responsible for the research questions and designed the study. LW, RvdM, EA commented on the design of the problem analysis and all authors commented on the design of the cRCT. EA is the team’s expert in economic evaluations. JS wrote the first draft of this manuscript and was responsible for the revisions. All authors critically reviewed the drafts and approved the final manuscript before submission.
